# Guilt, shame, anger and the Chicana experience: Cherríe Moraga’s *Native Country of the Heart* as voice of resistance

**DOI:** 10.1080/01440357.2020.1816874

**Published:** 2020-09-15

**Authors:** Mario Grill

**Affiliations:** Department of English, University of Klagenfurt, Klagenfurt, Austria

**Keywords:** Chicanx studies, emotions, shame, cognitive literary studies, narrative theory, memoir

## Abstract

Much scholarly attention has been paid to Latinx fiction. Less scholarship has focused on Latinx nonfiction, especially in the contemporary period. This essay focuses on the affective and political function of the Chicana memoir, particularly Cherríe Moraga’s *Native Country of the Heart* (2019). I explore how the emotions evoked by such a memoir aid in resisting dominant narratives of oppression. Counteracting such narratives of constraint and discrimination, Moraga creates a new conceptualization of empowering cultural imaginaries. I propose that the emotionalizing strategy of *Native Country* will provide new insight into how Chicana memoirs can function as and are voices of resistance against the marginalization of Mexican American women. Indeed, mentally and *emotionally* sharing such narratives might decelerate the constant fueling of a system of intersectional racism as *Native Country* exemplifies how even the memory of the unlettered can act as powerful means of resistance against the colonialization of the mind.

The United States may be known as melting pot, but it is also home to many forms of dehumanizing or cruel efforts to dehumanize certain (ethnic) groups. Indeed, being othered or being exposed to racial shaming are common practices. In the case of the Chicanx or Latinx community, Stephanie Fetta reminds us that “[a] flux of perceptions rather than a finite set of traits, racializing Brownness as a shameful form of being is a staple practice in many parts of the United States” (Fetta 10). By doing so, these racializer oftentimes rely on the use of negative, or sometimes referred to as “ugly”, emotions guilt, shame, and anger to severely harm others who are then, “are irrevocably shamed into a social stigma” (11). Seeking to understand how racialization works and how it affects those who are othered means to consider how racialization takes place and what these negative emotions do to the “victim”. In her *Shaming into Brown* (2018), Stephanie Fetta has extensively argued how these steps of racialization take place and how racial shaming works. In this context, she stresses that “[u]nderstanding the sociality of shame and other affects prevents the facile reduction on Latin@/x identity to an essentialized self” (Fetta 164).

Taking a cue from Fetta’s research, this piece is also interested in the sociality of these emotions. I want to add that it is also just as important to consider how narratives invite readers to mentally share and *feel* some of these “ugly” emotions and gain a better understanding of Chicanx life and culture as they mentally simulate how nonfiction texts may give voice to the voiceless and create spaces of solidarity and resistance. In the first part, I outline different theories on the emotions guilt, shame, and anger and explore how they manifest in Chicana memoirs. In the second part, I employ a narratological analysis of the emotionalizing strategy of Cherríe Moraga’s memoir *Native Country of the Heart* (2019). Here, I argue how transcending this host of negative emotion aids in co-shaping narratives of new memory despite the United States’ current bleak political climate. While racialization and dehumanizing efforts are “normal” parts of U.S. culture, I propose that imagining and *feeling* what Moraga and her loved ones were going through at the time might aid us in no longer fueling a system of intersectional racism and instead showcases how even the memory of the unlettered can act as a means of resistance against the colonialization of the mind.

The history of the Chicanx community within the highly racialized political climate of the United States is often represented as a narrative of oppression. And indeed, the community has been on the receiving end of countless negative stereotypes and continues to be subjected to various forms of economic, social, cultural, and political discrimination. Dating back at least to the early days of the Chicano movement there have been voices of resistance against these interlocking forms of oppression from within the Chicanx community, speaking through literary texts to create empowering counter imaginaries. Many of these imaginaries are fiction, but memoirs, too, have emerged as an important genre within this larger chorus. Unlike novels and short stories, these nonfiction accounts purport to relate events from the actual lives of their authors and thus serve an important political function. Norma Elia Cantú notes that Ernesto Galarza’s *Barrio Boy* (1971) and Oscar Z. Acosta’s *The Autobiography of a Brown Buffalo* (1972) are typical examples of Chicano memoirs that “reflect the social and political oppressions of their times” (Cantú 317). Already in these early texts, we can observe how Brownness is racialized in the United States. In addition, reading them today is a powerful reminder that the ruthless policies of the Trump Administration against the people who trace their ancestry back across the Mexican-American border indeed are steeped in a long history of exclusion, discrimination, and oppression.

There are, however, also forms of oppression *within* the Chicanx community, and they are more typically highlighted in memoirs authored by women. As Cantú points out, Gloria Anzaldúa’s *Borderlands/La Frontera* (1987), Mary Helen Ponce’s *Hoyt Street* (1993), and Ana Castillo’s *Massacre of the Dreamers* (1995) “all, in one way or another, address the subalternity of the writer’s lives” (Cantú 318), offering readers a glimpse into what it means to be oppressed not only for one’s belonging to the Mexican-American community but also for one’s gender and sexuality. Taking a cue from C. Alejandra Elenes' research (2000), Cantú proposes that we rather understand Chicana life-writings as collective autobiographies because for “many Chicana and Latina authors, writing personal memoir or autobiography constitutes an exercise in communal storytelling insofar as many of our stories cut across age, geography, and even gender and tell a shared story of injustice and prejudice” (Cantú 320). More recent texts such as Reyna Grande’s *The Distance Between Us* (2012) or Cherríe Moraga’s *Native Country of the Heart* (2019) further support Cantú’s argument as they, too, tell a shared story of injustice and prejudice that is shaped not only by American racial politics but also by the patriarchal and heteronormative values that still dominate the Chicanx community. And like other Chicana memoirs before them, both texts highlight the negative emotions – emotions such as guilt, anger, fear, and, above all, (racial) shame – that are triggered by the constant struggle against such traditional values and the desperate attempts to overcome them.

Moraga’s text is of particular interest for its complex treatment of these negative emotions and the ways in which it makes them accessible for readers. Cantú argues that whether a text is received as intended depends on whether a reader is part of an in- or an outgroup as outsiders might lack this collective experience and might consider the text as mere entertainment. In addition, we have to acknowledge that neurocognitive studies evidence that test subjects “notably alienate out-group members, making racialized perception an important factor in rapport and social acceptance” (Fetta 16). Texts like Moraga’s, however, are inviting readers mentally walk in unknown territory, free from prejudice. Indeed, “Latin@/x literature, and other marginalized literatures, allow us a unique opportunity to expose ourselves to the life experience of someone we often cannot perceive without narrow mental schemas and discriminatory scripts” (35). Thus, this literature mentally cues readers of all sorts, regardless of whether they belong to an in- or an outgroup and while it may be easier for someone to feel with someone from their own group, we should not overgeneralize. As Frederick Luis Aldama reminds us, “[a] Chicano reader may [also] be moved by a Chinese author – as well as by a Japanese author” (Aldama 47), simply because there are “narrative devices used by authors to move their readers with shared universal emotions” (47). Taking a cue from Aldama and drawing on the analytical tools of cognitive narratology, I will thus argue that even out-group readers may be emotionally affected by *Native Country of the Heart* when it invites them to share some of Moraga’s life experiences by mentally imagining and *feeling* what she and her loved ones were going through at the time. For in-group readers, however, these experiences may resonate differently, or even more strongly, because they have experienced similar things or know someone who has.

Telling the story of her mother, Moraga stresses how easily the life of a Chicana woman can be erased and what effect this might have on the following generations. Continuing her life-long efforts of being a voice of resistance, Moraga uses *Native Country* to actively oppose social scientist Arthur Rubel’s claim that a Chicana is “ideally submissive, unworldly, and chaste” (Rubel 214). In addition to rejecting this claim on the level of gender, Moraga has always resisted it on the basis of her sexuality. Aldama points out that back in the 1980s, she was among a “critical mass of queer Chicano/a artists and intellectuals” that “forced open the gates to an otherwise generally homophobic *raza*-nationalist (late 1960s and 1970s) political movement” (Aldama 21). In *Native Country*, Moraga counteracts narratives of oppression based on both gender and sexuality, as well as those collective narratives that highlight the constraints of Mexican womanhood and forced assimilation. Importantly, the memoir is also a testimony that the pursuit of finding one’s place and voice in the United States comes with a host of negative emotions including guilt, shame, and anger. In the age of movements such as #MeToo or #BlackLivesMatter, Moraga’s text might even more strongly resonate with both in-group and out-group readers.

## Guilt, shame, anger and the Chicana memoir

Feeling guilty or ashamed is a painful experience that most people have had at one point in their lives. As the cognitive narratologist Patrick Colm Hogan points out, guilt and shame are “self-blame emotions” that usually “involve a past action that is aversive and a spontaneous attribution of causality to oneself” (Hogan, *What Literature Teachers Us About Emotion* 216). It may also be possible to experience these negative emotions when someone anticipates future actions, but they only “occur in simulations where one imaginatively places oneself after the act” (216). While shame and guilt often appear together, there are important distinctions between them. Indeed, Psychologists Michl et al. stress that “[s]hame and guilt can be differentiated theoretically: While the feeling of shame implicates the presence of other people; guilt can arise and persist without others” (150). In other words, guilt is more commonly understood as a private emotion while the experience of shame is to a great extent influenced and shaped by others. In addition, “shame is more complex than feeling guilt” because “shame is not so much based on general social standards but rather on culturally independent social settings” (155). This “ugly” emotion can be quite severe, as it “is ultimately about punishment, is self-focused and ‘wired into’ the defense system” (Gilbert 1225) and can be focused on the self too. For example, I can feel ashamed of myself if I miss an important deadline and this experience automatically has me evaluate my own self negatively.

Shame also plays an important role in racism and in racialization as “we are socially scripted to perceive race” (Fetta 3). In her investigation of Latinx literature, Fetta demonstrates through the soma, “the intelligent, communicative body” (xiii), “how racialization specifically employs shame to give affective materiality to the physical notion of race” (xvi). Similarly, empirical data also confirm that “that shame is most often experienced in the presence of others” (Deonna, Rodogno, Teroni 23) and the intensity of this emotion actually increases depending on public exposure and to some degree on disgust. Several researchers, for example, Tangney, J. P. and Dearing, R. L. (2002) or Smith, R. H. et al. (2002) have shown in these degrees in their works. While shame and guilt are often portrayed together, guilt is distinctly different from the social emotion shame.

Guilt is “commonly ‘self-focused’” (Hogan, *What Literature Teachers Us About Emotion* 180) and predominantly focuses on the feeler and does not need to include others. Indeed, guilt “may be based less on predicting others’ reactions, and more on the culture-specific normative moral knowledge of right and wrong“ (Michl et al. 155). Similar to shame, guilt needs a clear understanding of the self, independent from others. Also, both emotions are social “in the sense that they typically arise in interpersonal context” and “guilt appears to motivate confession, apology, and reparation” (Tangney 543). While many people tend to use shame and guilt interchangeably, guilt is distinctively different as it is less a public emotion than shame. Feeling guilt is “typically a less painful and devastating experience” and “doesn’t affect one’s core identity. Instead, there’s a sense of tension, remorse and regret over the ‘bad thing done’” (546). While  shame is often called ones of the “ugliest” emotions, guilt is oriented toward a reparative behavior, less about defense, and more productive for the self. This may also be one reason why it is possible to address and to find redemption for guilt. The contrary holds true for shame as it is often “focused on an unalterable act of the past“ (Hogan 218) and one can overcome this negative emotion only through “achieving some parallel excuse, some compensatory success“ (218) but no reparation as is possible for overcoming guilt. As this shows, then, self-blame emotions such as shame and guilt can be caused by both the self and by others. Not only can they arise in response to one’s own transgressions and moral wrongdoing, but they can also be caused by one’s internationalization of blaming.

Again, this is particularly true for shame, precisely because of its sociality. Shame is considered a social emotion because the feeler “is, or behaves in a way that, she realizes, endangers her standing within a given social sphere” (Deonna, Rodogno and Teroni 30). Unfortunately, one can feel good about oneself or be a “good” person but one’s environment can still make one experience shame. If others keep blaming or shaming you for doing something wrong or punish you for being who you are, you might at one point internalize these feelings too. This can also happen if you are being shamed unfairly, if you did not cause a negative situation, and especially if you are exposed to racial shaming and racialization. Understandably, “shame is likely to go hand-in-hand with hostile and destructive behavior” and “distinctively associated with *depression*” and even “linked to decreased well-being and has obvious damaging consequences for constructive social interactions” (48). Considering how damaging an environment can be for someone, it is important to consider how such an environment can cause someone such a feeling. We can, for example, observe the full scope of how influential self-blame emotions can be in Chicana memoirs as I will argue in my analysis of Moraga’s *Native Country of the Heart*. Through this text, readers also witness “the burden of intersectional racial shame” (Fetta 5) but Moraga eventually resists such “habituated, socially cued behaviors” (7). Thus, this analysis aims to partially explore why feelings of guilt and shame have become inextricably intertwined with the personal and cultural memories of the Chicanx community. However, I also want to stress that the Chicanx community does not allow AngloAmerica to define them through forcing them into experiencing such negative feelings or through their ongoing dehumanizing efforts.

The long history of exclusion, discrimination, and oppression that has been endured by the Mexican American community is alive in people’s (emotional) memories, not least because they are constantly reminded of the fact that it is still a history in the making. Guilt and shame “may orient one’s attentional focus toward memories of the act itself. The memories, may, in turn, dominate working memory processes. This may lead to an experience of the derealization of the current, material world” (Hogan 217) (which is particularly like if there are no alternative ways for improvement). However, “[t]his derealization is particularly likely when no actional outcomes are available that would change the situation in a productive way” (217). A hostile and racializing environment may never allow for someone to feel otherwise as a racializer “shames the other into believing him or herself different and unworthy” (Fetta 7).

Similar to how guilt and shame are oriented toward one’s own memory of an act, so is the general assumption of emotional memories. These are implicitly produced memories of emotionally arousing experiences and when activated, an “emotional memory (roughly) leads one to re-experience the emotion” (Hogan 51). While episodic memories “bring representational content into working memory” (55), an emotional memory “may be activated without a corresponding representational memory” (55) and partially guides our expectations. Indeed, it is our own memories of certain events that may guide us toward feeling a certain way as we have already felt that way in a similar situation before. Indeed, “socialization is the development of emotional inclinations on the basis of emotional memories or cognitive processes that bias one’s emotional response” (Hogan Literature and Emotion 65). For example, U.S. Americans share some collective emotional memories of 9/11, members of the Latinx community share some collective emotional memories related to the U.S.-Mexico Border, and Europeans share some collective emotional memories of the Holocaust. This specific form of memory can be quite formative as well as enduring and may guide our emotional responses.

This also holds true for the development of internalized guilt and shame. While both emotions can lead to anger, more research on the emotion shame was been conducted. Indeed, in a clinical study, Helen Block Lewis (1971) “first formulated a theory of the links between shame and anger” which revealed that “patients’ expressions of anger and hostility were often preceded by manifestations of shame” (Deonna, Rodogno and Teroni 54). Lewis’ findings strongly suggest that when someone has been blamed and shamed and guilt-tripped for too long – and if they feel that they have been treated unjustly – they will, at some point, very likely develop another negative emotion in response to this injustice: anger.

The primal emotion anger can have many forms and, more often than not, carries negative connotations with it. Cognitive literary scholar Sue J. Kim argues in her *On Anger* (2013) that the concept of race is central to our conceptions and experiences of anger. Kim explains that anger “may be partly physiological, cognitive, and psychological, yet it is also deeply ideological, inseparable from factors such as race, class, gender, sexuality, ethnicity, nation, and religion” (2013 1). One specific form of this emotion, is the so-called racial anger, which “is and is not about race” (5). It “refers to the anger arising from racial/national formations appearing in various specific forms around the world, commonly conditioned by global capitalism, colonialism and neocolonialism, and liberal individualism” (5). It becomes evident that racial anger is also a reaction to injustices taking place just like current riots which are, again, about and not about race as it is inextricably linked to these social constructs.

However, anger and other emotions are also sometimes used by racializers to harm others. Indeed, “disgust, contempt, anger, and fear become affective capital used by racializers to claim harm done by the mere existence of another punishable by shaming” (Fetta 12) to emotionally deauthorize their targets. In addition, Fetta outlines that in “the case of intersectional racialization and racism” (13), responses of anger are considered wrong as they would challenge an oppressive cultural system (Kim even stresses that “a woman’s anger is anti-feminine” (1)). Indeed, the emotion anger, “marks the *feeler* as pathological, dangerous, or ignorant more sharply than do other emotions” (Kim 4).^1^ When we consider why someone experiences anger, we cannot overlook the fact that “anger also reflects conflicts within a society” (2). Anger, again, is not a solely negative emotion that might mark the feeler as dangerous, but also offers valuable insights into issues within a society.

For Chicanas, however, the experience and expression of all these negative emotions has been even more complicated because of the specific intra-communal oppression many of them have experienced. Often, in the context of Chicanx cultures, such intra-communal oppression is related to gender and/or sexuality, and Mexican-American women have to come to terms with it *in addition* to the other, extra-communal forms of oppression they also are subjected to.

Just like Chicanas continue to be subjected to various forms of discrimination, so are their literary productions. Christopher González explores in his *Permissible Narratives* (2017) why the works of Latinx literature have often been ignored. He points out that “[t]he aim of narrative theory has been to determine how narratives are made, how they work, and how they are consumed” (2) and these investigations should also include how or why narratives are able to emotionally affect readers. Here, González stresses that “to have narrative permissibility there must be an audience willing to engage with a variety of Latino/a writing” (3). He too does not make a distinction between readers from an in- or an out-group. Instead, González proposes that instead of employing an identity-based examination of this literature, we might do better considering how it functions in relation to literature more generally. Building on cognitive narratologist David Herman’s concept of a “storyworld” (Story Logic 2002), González suggests expanding it so “that it recognizes the sensorial and emotional factors of not only building a storyworld but inhabiting a storyworld as well. Readers dwell within the storyworld even as they reconstruct it from the text” (19). Indeed, through “narrative worldmaking” (17) readers take the narrated world and reconstruct it in their minds” (17).

Indeed, mentally reconstructing a certain situation generally helps to make sense of events or even our surrounding world and we do so through the so-called process of mental simulation. Simulation is, according to Hogan’s definition, “the imagination of particular conditions and particular, usually causal sequences of events without the constraints of perception and memory” (43). In addition, he considers this spontaneous, effortful, or implicit process “a key element in empathy and in literary understanding and response” (183). Drawing on Hogan’s findings, González stresses that simulation “can help explain how printed words on a page can evoke real emotions within a reader, even when a reader knows the narrative is a work of fiction (20)”. While readers certainly know that what they imagine is a simulation within their minds, we have to acknowledge that “the reader’s emotions are real” (20). Of course, the more immersed a reader is and the more vividly they mentally (re)create a storyworld, the more strongly are their emotional responses. In fact, readers “simulate, in part, what the author conveyed” and, ideally, “fill in what is left out of the text – often including, for example, unmentioned details of character appearance or intention” (Hogan Literature and Emotion 44). Ideally, readers do not differentiate between whether someone is part of an in-group or an out-group and allow themselves to share some of these emotional experiences. Indeed, “[t]he brain actually experiences events it is actually only reading about, and this power of simulation (mimesis, reliving) is an important basis of immersion” (Jacobs and Schrott 130).

Focusing on emotional experiences in my analysis, I want to stress how *Native Country of the Heart* invites readers to share some of Moraga’s life experiences by mentally imagining and *feeling* what she and her loved ones were going through at the time. Imagining even negative emotions – emotions such as guilt, shame, fear, or anger – is part of how readers mentally reconstruct the storyworld they dive into as they emotionally respond. In addition, “[t]he task of reading employs our minds in an embodied way” (Fetta 8), meaning that our brains *do* feel what we read about. In other words, *Native Country* might resonate strongly with in-group readers as they share some collective emotional memories while out-group readers are invited to witness this narrative and its emotional prowess. Indeed, a major asset of literary texts is that they “have the potential to alter our perceptions and teach us how to interpret unfamiliar phenomena (Moya 164). Thus, I will now turn my attention to *Native Country of the Heart* and explore how it is also a testimony that the pursuit of finding one’s place and voice in the United States comes with a host of negative emotions including guilt, shame, and anger. Even through “racializing Brownness as a shameful form of being is a staple practice in many parts of the United States” (Fetta 10), Moraga is one of many Chicanx (and Latinx) authors resist this form of oppression, hatred, and cultural practices. Accordingly, this narratological analysis of the emotionalizing strategy of *Native Country* will then provide us with new insight into how Chicana memoirs can function as and are voices of resistance against the marginalization of Mexican American women.

## Transcending negative emotions – the Chicana experience

Cherríe Moraga’s memoir *Native Country of the Heart* sheds light on the life of her mother Elvira as well as the painful experience of being a Mexican woman in AngloAmerica while losing her own memory. In addition, Moraga also explores how she struggled with being a lesbian Chicana in light of the constraints of the Mexican patriarchal system that further added to internal bias based on gender. Pressing on issues of cultural memory, shared narratives of migration, displacement, remembering, and having a sense of home again, *Native Country* functions as a creative tool and a counternarrative of resistance against AngloAmerica’s ongoing efforts discriminate and dehumanize Mexican Americans.

It is not new that Moraga has such a goal in mind as becomes clear when reading how she also reflects on her personal writing process. In the afterword to the fourth edition of *The Bridge Called My Back*, she discusses “those self-doubts, those deeply internal questions about our ‘right to write’” (249) and promises herself to not allow herself to succumb to these doubts. Similarly, Gloria Anzaldúa also wrote about the potential dangers of allowing oneself to be pushed to the margins of society, calling it a “life in the shadows” (20). Moraga, “*a white girl gone brown to the blood of my mother*” (Italics Moraga’s 10), also knows exactly how essential it is to resist those dominant modes of oppression and discrimination Chicanas face on a daily basis (She would later refer to this struggle as intersectionality). Indeed, she stresses that no aspect of a woman of color’s life “is wholly dismissed from our consciousness, even as we navigate a daily shifting political landscape” (xxii). Similar to how she narrates her mother Elvira’s life, women of color “have traditionally served as the gateways – the knowledge-holders – to those profoundly silent areas of expression and oppression” (xxi). Through many of her works, and most recently through *Native Country of the Heart*, Moraga – through pressing on the significance of even the memory of the unlettered – counters this effort of silencing, and demonstrates the importance of empowerment, both within Chicanx culture and within AngloAmerica.

Already in her first sentence, Moraga the narrator confesses to her readers that “Elvira Isabel Moraga was not the stuff of literature. Few bemoan the memory loss of the unlettered” (3). Instead of reading exclusively about Moraga, readers get insight into Elvira’s life and how easily her life and existence can be forgotten. These two sentences are quite important as Aldama reminds us in *A User’s Guide to Postcolonial and Latino Borderland Fiction* (2009) that “[t]he opening lines foreground the reader’s own processing of narrative’s coherence (logic system) and whether we invest in the story (emotive system) as a result of this coherence” (41). In these two sentences, Moraga sets the tone for the creation of new cultural memory against ongoing efforts of being silenced. Echoing Anzaldúa’s claim that “writing is a tool for piercing that mystery but it also shields us, gives us the margin of distance, helps us survive” (167), Moraga too thus creates such a narrative of survival. Doing so does not only allow the memory of Elvira to survive and to be recognized, but it also means that Moraga uses her nonfiction to reconnect to her indigenous heritage and to preserve her cultural roots. As Mary Pat Brady explains, “for Cherríe Moraga, memory, desire, and the body cannot be disentangled from each other; they call and respond to one another, shaping one another’s conditions of possibility. […] [s]he does not omit memory and desire from her analysis” (138). Importantly, Moraga “focuses on these subjects not as merely historical signs but because they are essential for surviving domination and exploitation: memory fuels resistance” (138). *Native Country* is a story of survival and Moraga foregrounds her and Elvira’s emotional experiences in this narrative. In the case of Elvira, a narrative of survival means to survive as a Chicana woman in a community strongly shaped and dominated by patriarchy. Sharing both positive and negative emotions with her readers, Moraga thus invites them to *feel* some of these women’s pains but also to mentally partake in their acts of resistance against ongoing efforts of silencing.

For Moraga, *Native Country* is not just her and her mother’s narrative, but importantly, their “personal record that testifies to a complex system of mixed-blood misnomered historical erasure” (238). Unfortunately, this systematic erasure is only one of the many examples of the dehumanizing and oppressing racial politics that govern the U.S. and further fuels acts of racialization and discrimination that also affect someone emotionally. As we have seen earlier, Chicanas have often been subject to internal bias or discrimination within the community, but they even receive more pressure from AngloAmerica. Such treatment certainly feeds negative emotions and causes someone to experience guilt or shame. Interestingly enough, while several researches have examined the interplay between guilt and shame, Fetta stresses that “it is not guilt but shame that structures and maintains U.S. race relations” (11). Moraga also explicitly outlines the whole scope of this complex system of historical erasure, as “disappear into Mexicanism is not enough; to disappear into Latinidad is even less of who we are; to disappear into Anglo-America, our colonization is complete. We are not supposed to remember” (238). Indeed, “Chicano/a culture represents one state of being (ontology) and/or way of knowing (epistemology), while the U.S. mainstream represents another completely different ontology and epistemology” (Hamilton 23), and through *Native Country*, Moraga exemplifies how dangerous and damaging this cultural difference is. The reason why Moraga or Elvira experience self-blame emotions is not because they choose to feel so but rather because they are forced to do so. In the case of shame in particular, the shamed may at one point “choose” to conceal that part of their identity for which they are shamed to somehow achieve compensatory success and no longer experience this “ugly” emotion. This is especially damaging in scenes of racialization as “the racializer establishes his authority (or Whiteness) and “affixes the other with social stigma – a permanent, socially definite attribute used to racialize a subject as an Other into social asymmetry” (Fetta 18). Indeed, as we learn in *Native Country*’s opening, this did not only happen for Elvira but instead for all Mexican American women of her generation (which may not come as a surprise for many readers). Elvira’s generation “was to disappear quietly, unmarked by the letter of memory, the memory of letter” (3). Being stripped of who they are and forced into experiencing racial shame for the remainder of their lives, this generation would leave no trace that they ever existed. Moraga even stresses that “when our storytellers go, taking their unrecorded memory with them, we their descendants go too, I fear” (3), especially because if we consider how “[w]e marginalize by ignoring the presence of the racialized” (Fetta 18). Already in the beginning, however, *Native Country* actively constructs a narrative that resists this form of oppression. Importantly, Moraga sets out to creating a narrative of new memory that resists the ongoing efforts of being marginalized or silenced.

Shame and guilt might not always necessarily be about an action one could have done to prevent feeling a certain way. As we have heard earlier, these emotions can also manifest themselves in the form of regret. Elvira’s narrative is “the story of our forgotten, the landscape of loss paved over by American dreams come true. And maybe that’s the worst of it (or what I fear) – that our dreams can come true in ‘America,’ but at the cost of a profound senility of spirit” (5–6). The American Dream may be the prime example for how much someone has to sacrifice in order to make these “dreams” come true. This does not only affect someone’s material possession but can also mean that someone has to leave their emotional belongings behind.

In an interview with L.A. Taco, Moraga stresses this second part, reminding us that “Indigenous cultures believe the land holds memory. In the dominant culture, that’s not valued, so we suffer from cultural amnesia as Mexicans in the United States” (LA Taco 2020). In addition to cultural amnesia, “[t]he American Dream means separation from ancestral, indigenous ways of knowing” (2020). Similar to many thousands of her generation, Elvira also experienced the Mexican immigrant experience, starting in 1925 “picking cotton in the Imperial Valley, just north of the California-Mexico border” (10). Instead of being able to concentrate on her formal education, Elvira and her sisters now had to work in the fields to support their families. Elvira, however, would almost be haunted by her strong feeling of regret. She “would never return to school after that. It had already become too embarrassing: a girl of eleven stuck into classrooms with third graders (11). This sacrifice Elvira had to make, would always leave an emotional harm in her.

Indeed, Elvira’s “bitterness (or better said: shame) about her lack of formal education was tacitly evident in every palsied signature she applied to grocery store checks, every job application my sister and I had helped her complete, every school notice we brought home to sit abandoned and unread on the kitchen table” (11). No matter where she went or what she did, Elvira could not escape her feeling of shame as her “inability to read and write well remained an open wound for Elvira her entire life” (11). While Elvira struggles with shame for the rest of her life, it has to be stressed that she had little choice in that matter and was socially forced to feel shame. Readers who can more easily identify with this young girl’s internal conflict are now too cued to mentally imagine Elvira’s hardship. In addition, readers’ own emotional memories are activated too as they might unknowingly remember their own writing experiences or how they would feel if they were in Elvira’s situation. In fact, we as readers “can become absolutely invested in a story because we feel (sometimes conflicting emotions) for a character and at the same time think beyond a character’s direct experiences; we can simultaneously feel sad and angry as well as formulate positive outcomes for the character” (Aldama 44). Not every reader has to agree with Elvira’s decision in order to share some of her feelings. While readers may even be cued to feel several, perhaps even conflicting, feelings about this decision, they nevertheless respond emotionally to her situation.

Her feelings of shame and guilt soon cause Elvira to be lost once she is institutionalized for her Alzheimer’s. Moraga even blames her own mother for being unable to decipher hospital signs as it is a sign of Elvira being powerless and silenced. Indeed, “*[h]ad she educated herself, she wouldn’t be in this situation, powerless among the gringos. […] Pendeja. That was what was wrong with her brain; not her memory, but education. Had she gone back to school, she coulda written her way out of this prison*” (Italics Moraga’s 155). The eleven-year-old Elvira was shamed in a way so powerful that shame would eventually “dominate [her] working memory processes” (Hogan 217). This open wound, caused by society, causes such a derealization in Elvira that she would not even seek for a solution to her problem. While Elvira could never escape this form of culturally caused shame, she did her best to make sure not to be a source of shame in one of her daughter’s most significant moments of her life. Instead, both women tell their personal stories of survival.

Chicana memoirs “tell stories of survival” (Cantú 322) and survival does not only happen on a physical level but can also be a mental conflict. For Elvira, survival refers to surviving as a Chicana woman in a community strongly shaped and dominated by patriarchy. Just like racialized feminist theory “addresses the problematic status of women of color as ‘the Other’” (Madson 3), Moraga too addresses this dominant issue in *Native Country of the Heart*. Her story of survival is one of many members of the LGBTQ+ community know only too well, the one to come out to family members, hoping the best and fearing the worst. Indeed, “[a] nagging uncertainty plagued me – about my womanhood, my sexuality, my mixed blood, ethnicity, all of which I had yet to fully acknowledge” (71–2). Within seconds, Moraga experiences all kinds of feelings as such a moment can change someone’s whole life. Here, readers are cued to co-create Moraga’s defining moment, calling on their own emotional memories of telling their parents life-changing news or of coming out themselves. She confesses that she felt that “[i]t seemed I would not, could not be loved. Not only because I was a lesbian, but also because, against my family’s wishes, I wanted to be free” (72). These intersectional feelings of guilt and shame now put Moraga on the crossroads and only intensify her desire to break free from these cultural chains. In addition, we should not consider Anzaldúa’s *Speaking in Tongue* (1980) that back then, “[t]he *lesbian* of color is not only invisible, she doesn’t even exist. Out speech, too, is inaudible. We speak in tongues like the outcast and the insane” (163). Moraga’s struggle here is not only a personal, but a collective and cultural and her narrative is proof of to find one’s voice to fill the silence a lesbian of color would be forced into for too long.

During her coming out to Elvira, Moraga the narrators invites readers to share her feelings and the pain of a possible negative outcome, while determined to no longer be treated like she is invisible. Although it is “just” a conversation with her mother (which can be one of the hardest for someone who wants to come out), Moraga immediately resists the general fact that “within seconds” (Fetta 28) people use information such as someone’s race, biological gender, or sexual orientation to “interpret the social power of the other” (28). Almost like she perceives her negative feelings of guilt and shame for being “different” as chains that bind her down, Moraga decides to break free from these imaginative chains and transcends these self-blame emotions by choosing that she is her highest priority. First, Moraga compares Elvira’s sounds on the phone to La Llorona and the latter asks her daughter “[h]ow can you get *satisfaction* from a woman?” (Italics Moraga’s 84). Instead of allowing Elvira to judge and effectively shaming her into being “wrong,” she stands by herself, affirming that “I had suffered too long and too hard for the right to love, and not even my mother was going to make me feel dirty for it. I would not abandon myself” (84). In general, “the process of coming out is different for everybody – where, when, how, the level of acceptance received from family and friends” (Bush 9). However, there are important aspect about this event that is shared collectively. Indeed, “the shared queer cultural schema allows for an understanding of these stories even if they do not exactly represent an individual’s experience” (9). The emotions Moraga feels at this way might now resonate very strongly with anyone who has had or experienced a coming out narrative, no matter where in the world they are. Instead of a “‘future anterior’ imagination” (216) which would give “the feelings of guilt, shame, and regret their deterrent function” (216), Moraga does not allow Elvira’s response to question her confession or to be shamed. Surprisingly, “[u]ntil she speaks, all the fury in her voice gone…,” Moraga writes, “‘[h]ow could you think that there is anything in this life you could do that you wouldn’t be my daughter?’ And that’s it” (84). Moraga’s story of survival here is presented in two ways. First, she does not even allow her mother to be the “author” of her feelings and chooses that her sexuality will not be the cause of self-blame emotions. Second, she creates important cultural memory by deciding that she does not let patriarchy hinder her from being free or to love who *she* wants to. However, she does not manage to break free from all constraints of Chicanx culture and the struggles caused by one’s one own family.

The Chicanx, or Latinx, *familia* is a highly complex unit and like many families around the globe, this unit too can be a safe haven or the cause of great psychological harm. Indeed, “as much as there is that unites members of the Latinx family, Aldama and González point out, “there are internalized ideologies that create divisions within the Latinx family” (60). Amongst those issues are those “that grow from internalized racism” (60) as well as “gender and sexuality structural striations” (60). While we have seen how sexuality can be an issue for Chicanxs, gender too is an issue in the Moraga clan. She confesses that “I do know that my sister and I were just plain guilty for being female, perhaps simply being females with hope; for feeling that we had a right to hope” (62). Instead of accepting that she is considered “worth less” than a man, Moraga soon starts to experience anger. While anger often focuses on the feeler, “[a]nger is dialectical. Understanding anger as dialectical means reading anger as an affective response to subjective and material senses of embodied frustrations” (Kim 6). Just like shame can endure and not be a singular experience, “anger can be understood as an ongoing (and not necessarily teleological) process” (6). This emotion too does not simply disappear and Moraga experiences it as the previously mentioned racial anger. Again, “[r]acial anger is and is not about race. Racial anger refers to the anger arising from racial/national formations” (5).

It is these formations and her upbringing in a racializing society that make Moraga feel this sensation. Early in the memoir, readers get a glimpse of this anger as Moraga the narrator blames Elvira for not demanding more of her life:“[m]aybe I was just done with the tears, with my mother’s inability to change, to give up the stories that caused her so much grief. Her refusal to stand up for herself and require a different life of her husband, of her never-satisfied mother, and, one day, of her son” (63). Wanting to be free herself, it does not come as a surprise that Moraga experiences racial anger about the facts that Elvira continued to allow others to push her to the margins of both their family and society. The Chicano experience in the Moraga clan, however, is the contrary to what Elvira and her sister had to endure. Their brother “James was the master of his own destiny. As females we would never know such freedom” (118). This also means that his position within the family unit would be the one with the most influence and weight. While the siblings argue about their father’s decision to postpone his hip surgery, “my father justified his inaction with ‘Your brother agreed,’ there was no mistaking the bitterness in my sister’s and my responses” (121). Here again, we can observe how strongly the Moraga clan is dominated and even guided by patriarchy, further fueling Moraga’s feelings of racial anger. While this situation shapes the family members’ relations to each other, Moraga also shares that in most “aspects of our cultura, James was, as he once described it, ‘just passing through’” (122). At the same time, however, “[m]y brother’s authority, as the eldest and a male, went unchallenged within our family” (122). Ironically, his behavior is the only aspect of his life in which he “remained inextricably Mexican” (122). It was Elvira “who had crafted my brother into the role of patriarch, the same role her elder brother and father had occupied in her own life. His long absences notwithstanding, my brother’s word was law” (122). Understandably, Moraga the narrator blames Elvira for giving James so much power over her and her family. Being further angered by her male family members, she recalls the moment she tells her father of Elvira’s passing. “When I tell my father,” she remembers, “the first words out of his mouth are: ‘It’s been so hard for me’” (222). Understandably, she admits that “I am disturbed that his *first* words are about him” (222) as it might appear not even her own death is about Elvira. Little by little, both her father and James fuel Moraga the narrator’s feelings of anger, but she again decides not to allow negative emotions to dominate her. At Elvira’s funeral, she holds a eulogy and “thanks” James “‘for finally saying last night at the Rosary vigil words my mother had waited a life-time to hear’”. After having said those few words, she observes how his face is “red with building rage,” and how, imaginatively, “I feel my brother, a stranglehold on my throat” (225). His reaction is only another instance that proves one thing to Moraga the narrator: “[t]he possession of the mestiza mother by the white patriarchs. This is what I felt for Elvira, looking out upon that funeral congregation” (229). Similar to her internal struggles with guilt and shame, Moraga the narrator here too does not allow a negative emotion to feed into this history in the making. However, she also confesses that, initially, “I could not profess what I knew to be so; that my mother was a woman of unyielding power that would not be suppressed” (229). Even though Elvira was subjected to a lifetime of discrimination, chains of patriarchy and racial shaming, this was and would not be how she would be remembered. Instead, “a memory spirit broke through me, and my eulogy became a rebellion” (229), honoring and cherishing Elvira for never giving up and to emphasize how her and her generation’s narratives are permissible and will not be silences. Indeed, Elvira’s narrative then becomes a conceptualization of empowering cultural imaginaries instead of disappearing quietly. Similarly, Moraga the narrator too turns her negative emotions into fuel of passion, and effectively filling the silence, giving a voice to the voiceless and by creating a space of solidarity and resistance.

This voice is deeply significant, since it also allows others to remember this person and their lives. Indeed, *Native Country* is a narrative that actively constructs “new memories and how these memories resist neocolonialism’s silencing narratives” (Irizarry 157). As could be observed through the depiction of both Elvira and Moraga the narrator hardships, such a narrative.

Indeed, such a narrative “combines experience, imagination, and agency to tell a new story of one’s identity, culture, or other community defining her or his belonging” (159). Closely dissecting their lives, Moraga makes it clear that neither she nor Elvira can be bound by the chains of old or oppressive for good. The one thing, however, that keeps on darkening Chicanxs lives, is, as identified by Moraga on the memoir’s final page: “[t]o disappear into Mexicanism is not enough; to disappear into Latinidad is even less of who we are; to disappear into Anglo-America, our colonization is complete. We are not supposed to remember” (238). Thus, her and countless other’s efforts to create acts of resistance are essential to fight those efforts. Moraga’s solution, for now, is the realization that “[t]here is no justification, but there is so much need for reckoning; for me and for Elvira, too. And in that reckoning, there is the need for return. […] I return through these pages” (238). *Native Country* is a narrative that invites readers to co-shape the storyworld of Elvira and Moraga the narrator. In this process of mental simulation, readers are cued to witness these Chicanas’ struggles of being (and surviving as) a Mexican woman in AngloAmerica. While they are forced to abandon their cultural roots and origins more and more, Moraga counters these efforts. Elvira’s narrative is proof of how for Moraga “memory fuels resistance” (Brady 138) as memories are an important source of empowerment. In addition, by inviting her readers to mentally imagine and partake in both Elvira’s and her own emotional struggles, readers are cued to share those emotions, effectively *feeling* with these Chicanas, and might even gain a better understanding of Chicanx culture and life. Ultimately, with Moraga stressing that she cannot be bound by chains of racism, gender, or even her own family, she effectively showcases how even the memory of the unlettered can act as a means of resistance against the colonialization of the mind.

## Conclusion

Chicanas are not only subjected to racialization and discrimination; they are also to some degree “guided” by internal issues within the Chicanx community. As Moraga powerfully displays in *Native Country*, that while such a narrative of injustice and prejudice comes with a host of negative emotions such as guilt, shame, and anger, memory fuels resistance. As Fetta pointed out, racism and “racializing Brownness as a shameful form of being is a staple practice in many parts of the United States” (10). However, Moraga, and Chicanas in general, resist such efforts of marginalization through the creation of counternarratives. In this case, Moraga presses on issues of cultural memory, shared narratives of migration, and having a sense of home again. While out-group readers might need more effort to mentally construct the storyworld of Moraga’s life, they too can mentally simulate what they read. Indeed, it is the experience of shame, guilt, and anger that might resonate with readers as these emotions can cue readers’ own emotional memories. While not having an identical experience, these emotional memories allow readers to relieve the feeling they had in a similar situation. Unfortunately, there may be hardly a person out there (especially if of color and in the United States) who has never felt the pressing weight of guilt, anger, or the sense of shame. Just like Moraga sets out to display in *Native Country*, these emotions do not have to govern someone’s life.

Functioning as a creative tool of resistance, *Native Country*, presses on issues of cultural memory, shared narratives of migration, displacement, remembering, and having a sense of home again. For Moraga, memory fuels resistance and her act of resistance is to highlight how the memory of the unlettered can give voice to the voiceless and allows them to relate their own narrative of survival. Many members of the Chicanx community can relate to the Chicano Migrant experiences and how many people (and often children) had to work in the fields. For Elvira, and countless other children, this would mean to discontinue her formal education and, as we have observed, cause her to be haunted by her sense of shame for the rest of her life. Indeed, her feeling of shame would become so powerful that she was never able to find “redemption” or some compensatory success. Moraga the narrator, by contrast, acknowledges how she is cued (and forced) to experience a host of negative emotions, in particular guilt, shame, and anger. She, however, transcends those imaginative chains and creates her own narrative of survival, free from patriarchy’s bounds. This struggle is not only a personal, but, as stressed by Moraga several times, a collective one that many Chicanas still have to fight these days. In addition, members of the LGBTQ+ community also share some emotional memories of their own narrative as someone who does not conform to heteronormative rules. As we have also seen, anger and its different faces are not only caused by a racializing society but one’s family can also be a feeding ground of this negative emotion. As we are reminded on a daily basis, Chicanxs and Latinxs continue to be on the receiving end of various forms of economic, social, cultural, and political discrimination, as AngloAmerica keeps on fueling a system of intersectional racism. *Native Country*’s emotionalizing strategy offers readers insight into this system that is so deeply entrenched in U.S. American culture. However, as Moraga has powerfully shown, she – and countless others – cannot be bound by chains of racism, gender, or even her own family. The Chicanx community may still be forced to experience a negative host of feelings. They are still continuously exposed to a host of negative emotions and often “shamed into Brown.” However, as Moraga has shown to us, even the memory of the unlettered can act as a means of resistance against the colonialization of the mind. This memoir, then, showcases how nonfiction texts can give voice to the voiceless, create spaces of solidarity and resistance, and fill the silence (queer) women of color were forced to inhabit for way too long and their fights for recognition are far from over.

Through *Native Country*, Moraga effectively offers readers insight into some of her (and her family’s) pain and invites them to share both her pain and her determination to create acts of resistance. Unfortunately, Moraga’s memoir is not the only example of how someone (or even a racializer) establishes authority to place a social stigma on someone to “Other [them] into social asymmetry” (18). On a daily basis, we can observe such practices all over the United States and in almost all cases, ethnic groups are on the receiving end of this dehumanizing efforts. If successfully ignored into marginalization, each one may disappear quietly, leaving no trace behind, as “[w]e are not supposed to remember” (Moraga 238). As I aimed to argue in this narratological analysis, Moraga constructs a narrative of “new memory and how these memories resist neocolonialism’s silencing narratives” (Irizarry 157). This memory and the prevention of gradual losses fuels resistance against narratives of oppression through empowering counter imaginaries that may lead us toward a new tomorrow that is not poised by “ugly” emotions that are used by oppressors, discriminators, or racializers to harm someone. Instead, Moraga offers a powerful example of how nonfictional narratives can act as voices of resistance against forms of oppression and marginalization.

## Note

1. Kim adds that „[r]ace, like anger, is ideologically and historically constructed, so many of the conflicts that seem to center on an essentialized concept of race are actually a collision of various historical-political factors; as we do anger, we fetishize race, or isolate it from the systems and structures out of which it arises (particularly economic systems)” (5).
